# Commercial versus synthesized polymers for soil erosion control and growth of Chinese cabbage

**DOI:** 10.1186/2193-1801-2-534

**Published:** 2013-10-17

**Authors:** Sang Soo Lee, Scott X Chang, Yoon-Young Chang, Yong Sik Ok

**Affiliations:** Department of Biological Environment, Kangwon National University, Chuncheon, 200-701 South Korea; Department of Renewable Resources, University of Alberta, 442 Earth Sciences Building, Edmonton, AB T6G 2E3 Canada; Department of Environmental Engineering, Kwangwoon University, Seoul, 139-701 South Korea

**Keywords:** Biopolymer, Erosion control, Plant growth, Polyacrylamide, Water quality

## Abstract

Soil erosion leads to environmental degradation and reduces soil productivity. The use of anionic polyacrylamide (PAM) and synthesized biopolymer (BP) using lignin, corn starch, acrylamide, and acrylic acid were tested to evaluate soil erosion, water quality, and growth of Chinese cabbage (*Brassica campestris* L.). Each treatment of PAM and BP was applied at 200 kg ha^-1^ to loamy sand soil and subjected to a slope of 36% with a 20 mm h^-1^ simulated rainfall. Application of BP decreased soil pH compared to the untreated check (CK); however, the soil pH was not altered with PAM. The decrease in pH might most likely be due to availability of anionic sites to be protonated on soils having pH >6 and soil buffering capacity. Both PAM and BP applications may not induce eutrophication with stable levels of total contents of N and P. With PAM and BP, the average values of suspended soil (SS) and turbidity were reduced by up to 96.0 and 99.9%, respectively, compared to CK. Reduction of SS can be attributed to increasing soil stability and shear strength by clay flocculation. There was no toxicity effects resulting from germination tests and the dry weight was increased by 17.7% (vs. CK) when PAM and BP were applied. These results are attributed to increases in water retention and plant-available water. The use of polymeric soil amendments is an environmentally friendly way to mitigate soil erosion and nonpoint source pollution.

**Electronic supplementary material:**

The online version of this article (doi:10.1186/2193-1801-2-534) contains supplementary material, which is available to authorized users.

## Introduction

Soil erosion is a natural process resulting primarily from forces of water and wind. Soil particles are detached from soil aggregates and moved to other locations by natural processes (McIntyre 1958; Troeh et al. 2004). Many factors such as the slope, soil properties, rainfall intensity, and management practices directly affect the yield of soil erosion or its acceleration rate (Agassi et al. 1990; Gerits 1990; Kinnell 2000; Troeh et al. 2004).

The slope is one of the most important factors determining the characteristics of soil erosion, particularly in highland areas which are located at 600 m above sea level (Agassi and Ben-Hur 1992; Polyakov and Lal 2004; Heo et al. 2008; Lee et al. 2011). Soil erosion can be multiplied without vegetation cover mainly due to the impacts of gradient (Kinnell 2000; WSDOT 2008). Many studies have shown that an increasing slope intensifies soil erosion, resulting from decreasing water infiltration rate, reducing potential of water ponding, and increasing velocity of surface water flow (Agassi et al. 1990; Bradford and Foster 1996; Fox and Bryan 1999). Therefore, soil erosion control in highland areas has received great attention. In Korea, the cultivable highland areas occupies greater than 10.4% of the total upland area or approximately 7.4 × 10^4^ ha (Partab 2004). Due to a high potential of soil erosion and a low temperature condition in highlands, a very limited number of plant species is being cultivated such as Chinese cabbage (*Brassica campestris* L.) and radish (*Raphanus sativus*) (Jung et al. 2005).

Soil erosion also causes nonpoint source pollution severely degrading water quality and aquatic ecosystems (Gantzer et al. 1990; Thompson et al. 1991; Mikkelsen 1994; Pimentel et al. 1995; Nagle 2001). Nonpoint source pollution induces exceed transportation of organic/inorganic nutrient chemicals from agricultural fields, thereby inducing eutrophication in aquatic systems and subsequent environmental devastation (Clark et al. 1985; Myers 1993; Choi et al. 2000; USEPA 2004; Shin 2006). Consequently, it threatens agricultural sustainability and requires enormous restoration costs (Clark et al. 1985).

Many conservative structures have been widely constructed to mitigate detrimental damages of soil erosion using natural or artificial materials (Nagle 2001; Lentz and Sojka 2009). At agricultural fields, the best management practices (BMPs) including mulches, vegetative buffer strips, contour farming, grassland formation, perennial plant cultivation, no-till cultivation, cover crops etc. were suggested (Troeh et al. 2004; Yang et al. 2006). Since the 1990s, the application of polymeric soil amendments has been known as one of the BMPs by many researchers and governmental institutes (Sojka et al. 2007; WSDOT 2008).

Anionic polyacrylamide (PAM) as a representative is an environment-friendly polymeric soil amendment for controlling soil erosion and runoff in the United States and Canada (Entry et al. 2002; Lentz et al. 2002; Al-Abed et al. 2003). The PAM refers to a broad class of compounds having a high molecular weight of 12–15 Mg mol^-1^ and functional group substitutions with different formations (Sojka et al. 2007). Charge density of PAM is typically 18%. Several hundred types have been synthesized with different types, numbers, and lengths of functional groups (Shainberg and Levy 1994; Entry et al. 2002). It should be noted that an anionic form of PAM is widely used because of its lower toxicity compared to other forms (Shainberg and Levy 1994; Flanagan et al. 1997; Sojka et al. 2007). PAMs contain less than 0.05% residual acrylamide monomer (AMD) for humans or aquatic species safety. For the economic aspect, the total cost of PAM application can be recovered by profit of increasing crop yield. Total cost of PAM application may reach to $106 USD ha^-1^ y^-1^ ($9.90 kg^-1^ PAM); however, the yields of bean and corn were increased by 12.3 and 4.8%, respectively, for a PAM-treated field compared to an untreated field. Lentz and Sojka (2009) insisted that increases of crop yield were sufficient to countervail PAM application expense.

Surface application of PAM to soil alters soil structure via clay flocculation (Quastel 1954; Orts et al. 1999,2007). Calcium as one of the electrolytes reduces the diffuse double layer surrounding soil particles and facilitates the bridging between soil particles and PAM molecules (Theng 1982; Fuller et al. 1995; Orts et al. 2000; Entry et al. 2002). The application of PAM has been known to stabilize soil aggregates (Sojka et al. 2007), improve soil aeration (Quastel 1952; Lee et al. 2008), and increase water permeability or infiltration (Mitchell 1986). McElhiney and Osterli (1996) found that the infiltration rate increased by up to 40% in sandy loam soils when a 0.01% PAM solution was applied. The use of PAM also reduces water turbidity and increases its quality (Flanagan et al. 1997; Ajwa and Trout 2006). For the PAM application in the fields having a relatively high slope, it has been known as an effective way to control soil erosion. Agassi and Ben-Hur (1992) conducted an erosion experiment using PAM and phosphogypsum (a source of Ca^2+^ as an electrolyte). They found that a combination of 20 kg ha^-1^ PAM with 10 Mg ha^-1^ phosphogypsum was very effective in erosion control on steep slopes ranging from 30 to 60%. Sepaskhah and Bazrafshan-Jahromi (2006) showed that a 4 kg ha^-1^ PAM application may be sufficient in controlling soil loss on loam soils with relatively flat slopes of 5–7.5%. Otherwise, a study of Lee et al. (2011) suggested that the amount of applied PAM should be increased as the slope increases for efficient soil erosion control. They found that application of 40 kg ha^-1^ PAM on 40% sloping soils reduced soil erosion by up to 72% compared to the untreated soil. A higher amount of PAM may be more effective in reducing soil erosion than a lower rate of PAM at ≥ 20% slopes.

Polymeric soil amendments increase seed germination. Chan and Sivapragasam (1996) found that PAM application at a rate of 7 kg ha^-1^ improved cotton germination rate by up to 84%. Sivapalan (2002) conducted a similar pot experiment using cotton seeds subjected to different rates of PAM at 0, 0.001, 0.005, and 0.01% by dry soil weight of Alfisol. They found that PAM increased seed germination through reducing soil penetration resistance. Seed germination also can be improved through the prevention of washing-away, and stimulation of seedling emergence or sward establishment (Roa-Espinosa 1996; Roa-Espinosa et al. 1999; Sojka et al. 2003).

Under drought or no irrigation condition, an increase of plant growth on the soils treated with PAM has been reported, mainly due to increasing plant-available water in soils (Johnson Johnson [Bibr CR29][Bibr CR30]; Cook and Nelson 1986; Woodhouse and Johnson 1991; Hüttermann et al. 1999; Sharma 2004). Studies of Johnson ([Bibr CR29][Bibr CR30]) and Woodhouse and Johnson (1991) also showed an increase in plant-available water when applied PAM or starch copolymer with sufficient electrolytes. Woodhouse and Johnson (1991) revealed that the use of starch copolymers increased growth rates of lettuce and barley, and improved plant survival in a silica sand during a drought period. Hüttermann et al. (1999) supported their studies and found that Aleppo pine (*Pinus halepensis*) seedlings survived up to 82 d with PAM application. It was 33 d longer than the untreated soils. They explained that soil water retention increased exponentially with increasing concentrations of superabsorbent hydrogel (Stockosorb K400) which indicates a cross-linked form. Ghehsareh et al. (2010) also found that soil water retention was increased with a 1% superabsorbent polymer (superab-A 200), thereby improving the growth rate of Ficus tree (*Ficus benjamina* L. ‘Starlight’).

More recently, a synthesized biopolymer (BP) was recognized as an alternative to conventional chemical-based tackifiers such as PAM. Natural and industrial by-products may be sources of BPs synthesis (Sojka et al. 2007; Liu et al. 2008). The BPs provide a cost advantage and an additional safety to environments by using natural and non-toxic materials such as lignin, starch, sugar, cellulose etc. (Parker and Ring 2005; Sojka et al. 2005,2007). Woodhouse and Johnson (1991) partially insisted that the effects of BPs on plant growth would be similar or better than the use of PAM. However, the effectiveness of BP for soil erosion control and its safety have not been clearly defined relative to that of PAM. Therefore, the objective of this study was to evaluate the effectiveness of PAM and BP applications on soil erosion control, water quality enhancement, and growth stimulation of Chinese cabbage.

## Materials and methods

### Soil

Soil was collected from the surface to a depth of 30 cm after eliminating vegetation in Haean-myeon, Yanggu-gun, Gangwon province, Korea (38°15’54” N lat., 128°07’02” E long.). The properties of soil are shown in Table 1. The soil was air dried and passed through a 2-mm sieve. Soil pH and EC were measured with a solution of 1:5 soil:water (Orion 3 Star, Thermo, USA). Soil aggregate stability and water retention were determined using an aggregate analyzer (DIK-2001, Daiki Rika Kogyo, Japan) and a pressure membrane extractor (1500F1, Soil Moisture Equipment Corp., USA), respectively (RDA-NIAST 1988). The organic matter (OM) content was determined by the Walkley Black method using an ultraviolet absorption spectrophotometer (UV-1800, Shimadzu, Japan), and the available phosphorus were analyzed at wavelengths of 610 and 660 nm by the Kjeldahl and molybdenum blue colorimetric methods, respectively (Bremner 1996; Kuo 1996; Nelson and Sommers 1996; Yoon et al. 2004). Contents of inorganic N were analyzed using an ion analyzer after 2-mol L^-1^ potassium chloride extraction. Exchangeable cations were determined with a 1 N ammonium acetate (NH_4_OAc) solution using inductively coupled plasma (ICP) (Sumner and Miller 1996; Ok et al. 2007). Additionally, the soil particle distribution was determined using the hydrometer method by Sheldrick and Wang (1993). Soil characterization was triplicated and the measurements of soil aggregate stability and water retention were done with five replicates.

**Table 1:** **Chemical properties of soil and treatments**

Treatments	pH	EC^†^	OM^‡^	Avail. P	NH^+^_4_-N	NO_3_--N	Exchangeable cations			
							Ca^2+^	K^+^	Mg^2+^	Na^+^
		dS m^-1^	%		mg kg^-1^			cmol kg^-1^		
CK	7.1 a	0.021 ns^§^	2.2 c	1129 ns	139 ns	20.4 b	4.3 ns	0.3 a	1.3 a	0.1 a
PAM	7.0 ab	0.022 ns	3.1 b	1349 ns	95 ns	20.9 b	4.0 ns	0.1 c	0.9 b	0.1 a
BP	6.9 b	0.031 ns	3.3 a	1226 ns	106 ns	31.9 a	4.1 ns	0.2 b	1.0 b	0.1 a

The same letters in table indicate no difference determined by the Tukey’s HSD test at a significance level of 0.05 (*n* = 3).

^†^ Electrical conductivity.

^‡^ Organic matter.

^§^ Not significant.

### Polyacrylamide and synthesized biopolymer

A commercial Soilfix G1 anionic PAM (Ciba Chemical Co., Germany) and a synthesized BP based on lignin, corn starch, acrylamide, and acrylic acid were used in this study. Characterization of the properties of each treatment were triplicated and shown in Table 1. To synthesize BP, a total of 4-g lignin, 0.4-g corn starch, and 16-g acrylamide were completely homogenized with 100 mL of distilled water having an electrical conductivity (EC) of 4.17 × 10^-4^ S m^-1^ and pH of 7.2, and then 12-mL acrylic acid was added (Liu et al. 2008). The solution pH was adjusted to 7.5 by adding sodium hydroxide (NaOH) and was dissolved with 200 mL of distilled water. At a temperature of 70°C, 1.2 g of potassium persulfate (K_2_S_2_O_8_) was added after 30-min stirring for the radical polymerization reaction. The BP was extracted using acetone ((CH_3_)_2_CO) and then dried at a temperature of 60°C. A granular type of synthesized BP was ground using a laboratory cutting mill (Ika M20 Labortechnik Staufen, Germany). Total nitrogen and carbon (TC) were analyzed using an elemental analyzer (Carlo Erba 1500, Carlo Erba Instruments, Italy).

### Simulated rainfall experiment

Soil was repacked into soil test beds (40-mm wide by 443-mm long by 78-mm deep) with a bulk density of 1.1 Mg m^-3^. Each 200 kg ha^-1^ (or 0.35 g) of PAM and BP was dissolved with 500-mL distilled water (EC 4.17 × 10^-4^ S m^-1^, pH 7.2) and was applied to the soil surface using a hand sprayer. The untreated check (CK) was also prepared with the same amount of distilled water five days before each experimental run.

Simulated rainfall with intensity of 20 mm h^-1^ was applied for 100 min to each treated soil test bed with a slope of 36%. The total N (TN) and total P (TP) in runoff were analyzed by the ascorbic acid reduction method, namely the Standard Methods for the Examination of Water & Wastewater (APHA 1995), using an ultraviolet absorption spectrophotometer (UV-1800, Shimadzu, Japan). Suspended solid (SS) in runoff was weighted by the glass-fiber-filter-paper method (APHA 1995) and the turbidity was determined by a turbidimeter (2100P Turbidimeter, HACH, USA). A scanning electron microscopy (SEM; S-4300, Hitachi, Japan) were employed to scan the morphological characteristics of soil particles from soil test beds with/without treatments. The characteristics of runoff were determined in triplicate.

### Seed germination experiment

A seed germination test was conducted in five replicates to examine the toxicity of PAM and BP to Chinese cabbage (*Brassica campestris* L.). Chinese cabbage seeds were washed twice using sterilized distilled-water and were also surface-sterilized with 1% sodium hypochlorite (NaClO) for 15 min. Each of 20 seeds was placed in a Petri dish and then the PAM and BP solutions at the concentrations of 0.05 and 0.1% were applied to moist filter papers in sterilized Petri dishes. The same amount of distilled water was also applied as CK. Three replicates were employed. During germination test, the incubation temperature was maintained at 25±2°C (ISTA 1996; Kim et al. 2010).

### Pot experiment

The pot experiment was carried out in a glass-topped shelter house located at the Gangwondo Agricultural Research and Extension Services, Chuncheon-si, Gangwon province, Korea (37°56’13” N lat., 127°47’08” E long.). Soil was packed into a Wagner’s pot with a size of 1/5,000 a and Chinese cabbage seedlings (30 d after germination) were transplanted into the pots. To avoid any nutrient effect, conventional fertilizers (N, P, and K) were evenly applied based on the RDA-NAAS recommendation (RDA-NAAS 2006). As mentioned before, the same amount of PAM and BP solutions were applied to the respective test pots. Chinese cabbage plants were sampled after 56 d. Dry weight after 48 h at 70°C, leaf length, leaf width, number of leaves, and chlorophyll concentration (in SPAD value; SPAD 502 Meter, Minolta, Japan) were determined.

### Statistics

Data was analyzed using one-way analysis of variance (ANOVA) with dependent variables of germination rate, growth indices including leaf length, leaf width, number of leaves, dry weight, SPAD value, and soil properties. Differences in amendment means were compared using Tukey’s honestly significant difference (HSD) test at a significance level of 0.05 (SAS 2003).

## Results and discussion

### Anionic polyacrylamide (PAM) and biopolymer (BP)

**Figure 1:**
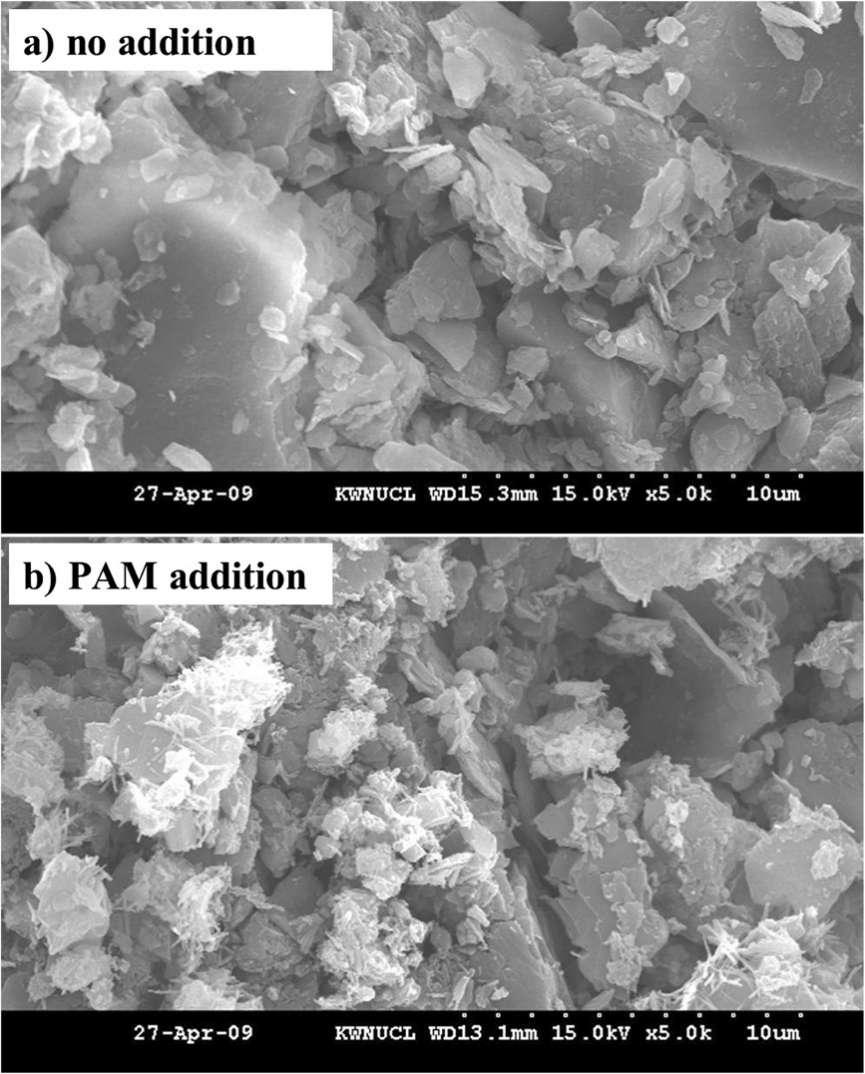
**Scanning electron microscopy (SEM) images: a) no addition and b) 200 kg ha** ^**-1**^ **PAM addition.**

To ensure changes in soil aggregates and pore characteristics in soil treated with PAM, the SEM images were produced from 200 kg ha^-1^ PAM-treated and untreated soils (Figure 1a,b). Images clearly showed that PAM addition stabilized soil structure and enhanced pore continuity. Similarly, Entry et al. (2002) and Sojka et al. (2005) used SEM images to report strand-like PAM binding to soil particles and concluded that PAM stabilizes the soil-surface structure and improves pore continuity in the soils.

The functional groups of the BP were analyzed at a wavelength range between 500 and 4,000 cm^-1^ using FT-IR spectroscopy (data not shown). Peaks at 1,558.54 and 1,401.05 cm^-1^ were assigned as ester groups (−COO^-^), and a peak at 1,653.64 cm^-1^ indicated a carboxyl group (C=O). These findings supported the idea of Liu et al. (2008) that these functional groups may improve soil aggregates with strong interactions between soil particles and BP molecules, thereby reducing soil loss. Our results were similar to their findings that the peaks of the ester and carboxyl groups were detected at 1,560 and 1,410 cm^-1^, and at 1,680 and 1,450 cm^-1^, respectively.

### Changes of chemical properties in soils

Application of BP decreased soil pH compared to the untreated loamy sand or the CK (*P* < 0.05; Table 1); however, no difference in pH was found between the CK and PAM-treated soil. When PAM or BP is applied to the soil, the acrylic acid units would be anionic when the soil is above pH 6 (Halverson and Panzer 1980; Sojka et al. 2007). Generally, these polymeric treatments decrease soil pH when initial soil pH > 6 because of the difficulty for anionic sites to be protonated and soil buffering capacity. No treatment effects on EC were found in treated soils. The soil OM contents in soils treated with PAM and BP were increased by 29.0 and 33.3%, respectively, compared to the CK (*P* < 0.05 for all cases). This phenomenon can be explained with the OM holding capacity of polymers having strong interactions between soil particles and polymer’s molecules in the soil aggregates during rainfall event. This finding agreed with studies of Dzhanpelsov et al. (1984) and Sojka et al. (2007) showing the improvements of soil physical properties and the stabilization of organic fractions in the soil when PAM were applied. Application of BP also likely increased soil OM and nitrate (NO_3_-N) in the soil as compared to the CK because it contains lignin and starch.

### Changes of chemical properties in runoff

The values of pH and EC in runoff from soils treated with PAM and BP did not differ with those from untreated soil or CK (*P* > 0.05; Table 2). Our findings revealed that both the PAM and BP applications would not harm the environment because the pH and EC values were within the safety criteria for normal water pH (6.05 to 8.50) and normal EC values (< 0.1 S m^-1^ in rivers and lakes) (WHO 2008). Shainberg et al. (1990), and Lentz and Sojka (2009) reported an increase in EC when PAM was applied to irrigation water. We partially agree with their findings and insist that the applications of PAM and BP may increase EC, but these are negligible.

**Table 2:** **Characteristics of runoff from soils treated/untreated with PAM and BP at each rate of 200 kg ha** ^**-1**^

Texture	pH	EC^†^	TN^‡^	TP^§^	SS^¶^	Turbidity
		10^-4^ S m^-1^		mg L^-1^		NTU
CK	6.87 ab	0.060 a	2.714 b	0.118 ab	9647 a	412.0 a
PAM	6.93 b	0.069 a	3.291 a	0.136 a	210.7 b	13.2 b
BP	6.64 a	0.075 a	2.740 b	0.090 b	429.2 b	14.8 b

The same letters in table indicate no difference determined by the Tukey’s HSD test at a significance level of 0.05 (*n* = 3).

^†^ Electrical conductivity.

^‡^ Total nitrogen.

^§^ Total phosphorus.

^¶^ Suspended soil.

With the PAM treatment, TN value in runoff increased by 17.6 and 16.7% when compared to the CK and BP, respectively (both *P* < 0.05); however, no difference in TN was found between the CK and BP. Our findings agree with previous studies suggesting that nutrient loss from BP-treated soil to aquatic ecosystems may be smaller than the soil treated with the same rate of PAM, thereby reducing the potential for eutrophication that is caused by inflow of nutrients or agricultural chemicals due to runoff and soil erosion (Lentz et al. 1998; USEPA 2004; Sojka et al. 2007). Results also showed that a large amount (200 kg ha^-1^) of PAM and BP applications may not influence surrounding ecosystems regarding to TN and TP concentrations. For the effects of PAM application on nutrient loss from soils, many studies showed that the surface application of PAM reduced nutrient loss because of reductions in runoff and soil loss (Wallace and Wallace 1986; Lentz and Sojka 1994 Lentz and Bjomeberg 2001; Entry and Sojka 2003; Sojka et al. 2005). Lentz and Sojka (1994) found that losses in N and P from topsoil were reduced by 83 and 84%, respectively, in response to the addition of PAM to the irrigation water. In this study, however, a higher TN concentration in runoff with the PAM was observed compared to BP. It may be explained with a higher N content of PAM (16.2%) compared to the BP (5.3%). Similarly, Kay-Shoemake et al. (1998) showed that PAM-treated soil contained significantly higher concentrations of NO_3_ and NH_3_ than the untreated potato field.

The amounts of TP in runoff from soils treated with PAM and BP were similar to the CK (each *P* > 0.05). However, with comparison in PAM and BP, the amount of TP in runoff from soil treated with BP was 28.6% lower than that with PAM. Both applications of PAM and BP treatments are satisfied with a safety level of TP in runoff, suggesting no detrimental effect on the environment. A higher level of PAM application, 200 kg ha^-1^ used in this study, may not deteriorate water quality and maintain the safety criteria of drinking water. We partially agree with a study of Lentz et al. (1998) that showed soluble P was reduced by 50% in Idaho’s Snake River Valley when PAM was applied. Goodson et al. (2006) also found that PAM application at concentrations of 1 mg L^-1^ and 10 mg L^-1^ reduced particulate P in tail water by 31 and 78%, respectively, and the higher level of PAM application significantly reduced TP compared to lower levels of PAM application or no amendment. Additionally, they mentioned that a reduction in soluble P depends on water salinity.

The suspended soil (SS) in runoff from soils treated/untreated with PAM and BP were determined. No difference in SS was found between PAM and BP (*P* > 0.05). Average SS values from the soils treated with PAM and BP were decreased by 96.0% compared to the CK (*P* < 0.05). We found that both applications of PAM and BP similarly increased soil stability and shear strength by clay flocculation between soil particles and molecules of these treatments. We suggest that the use of BP can be an environmentally friendly alternative to PAM that has extensively researched as a soil amendment (Theng 1982; Wallace and Wallace 1996; Zhang et al. 1998;2006; Tang et al. 2006). Orts et al. (2000) showed that the BP suspension made from microfibrils of cellulose, starch xanthate, chitosan, and acid-hydrolyzed cellulose microfibril, reduced soil sediment by an average of 80% compared to the CK. Application of the BP treatment should be practically used to not only reduce raindrop impacts such as soil-surface seal formation, soil detachment, and degradation of water quality, but also reproduce industrial wastes (Levin et al. 1991; Orts et al. 1999,2000,2007; Lee et al. 2008; Liu et al. 2008).

The turbidity was significantly decreased in response to PAM and BP applications compared to the CK (*P* < 0.05). No difference in turbidity was found between PAM and BP applications into soils. However, the average turbidity with these treatments was 99.9% lower than the CK. Our findings agree with studies showing that polymeric soil amendments improve water permeability and interfere with detachment of soil particles, thereby stabilizing soil aggregates and structures (Deery et al. 2002; Entry et al. 2002; Al-Abed et al. 2003; Goodson et al. 2006; Orts et al. 2007; Sojka et al. 2007). Al-Abed et al. (2003) found that turbidity was reduced by an average of 60% with PAM treatment at concentrations of 5, 10, and 20 mg L^-1^. Goodson et al. (2006) similarly revealed that PAM applications at concentrations of 1, 5, and 10 mg L^-1^ produced significant reductions in irrigation water turbidity by 73, 82, and 98%, respectively. From significant reductions in turbidity using PAM and BP, we suggest that the use of polymeric soil amendments on sloping non-vegetative or bare sites such as agricultural highland areas after harvesting effectively reduces runoff and soil loss, allowing decreasing nutrient loss increasing crop productivity.

### Aggregate stability and water holding capacity

**Figure 2:**
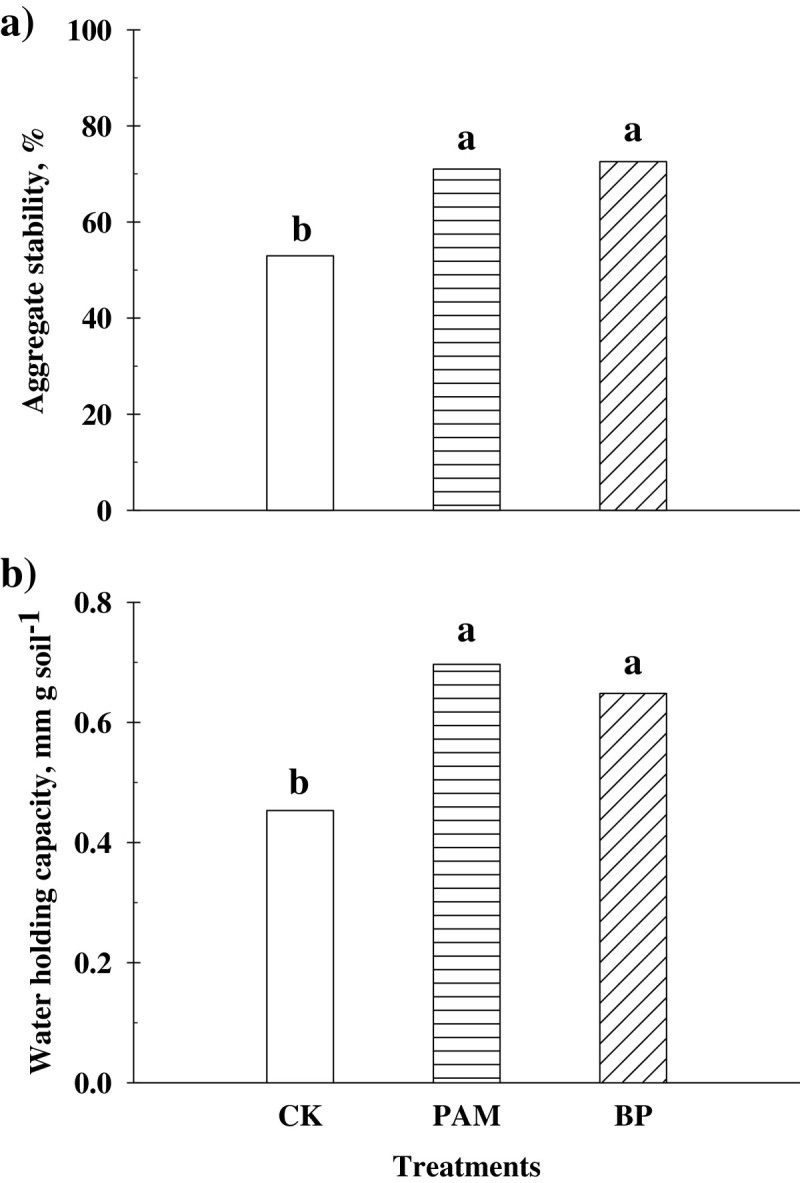
**a) Soil stability and b) water holding capacity (** ***n*** **= 5).**

Aggregate stability was increased by 25.4 and 27.1% for soils treated with PAM and BP, respectively (*P* < 0.05; Figure 2a), compared to the CK. An increase of aggregate stability in soil treated with BP was 2.2% higher than in soil treated with PAM, but was not significant. We found several possible reasons from previous studies indicating that the applications of PAM and BP induce clay flocculation in presence of Ca^2+^ which shrinks the electrical double layer and bridges PAM molecules and soil particles, thereby stabilizing soil aggregate structure (Wallace and Wallace 1996; Orts et al. 2000; Sojka et al. 2007).

For soils treated with PAM and BP, the water holding capacity was increased by an average of 32.6% compared to the CK (Figure 2b). Application of PAM has been found to effectively increase water permeability due to increased porosity and soil aggregation (Bouranis 1998; Kim et al. 1998). Other physical parameters such as water infiltration and retention, aeration, water drainage, and resistance to soil compaction and crusting may be increased in the soils treated with polymers (Cook and Nelson 1986; Zhang et al. 1998; Lee et al. 2008).

### Germination test for Chinese cabbage

**Figure 3:**
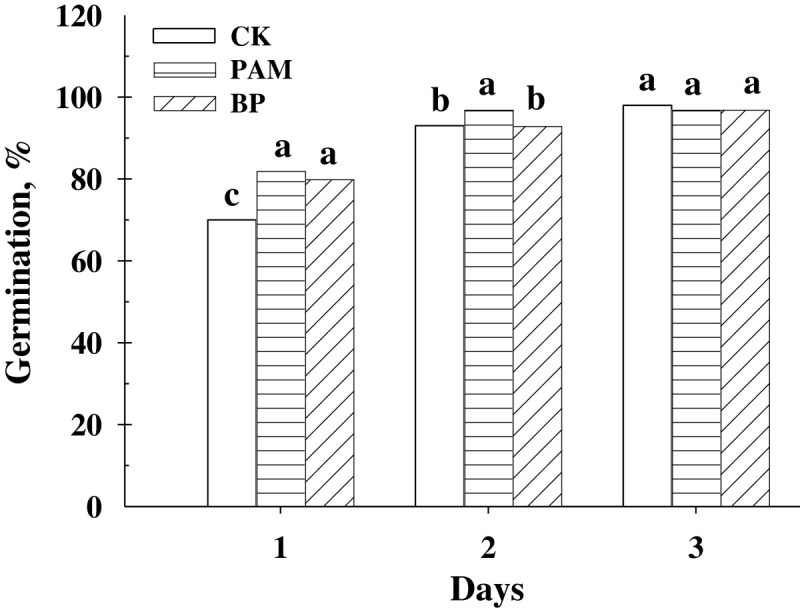
**Germination rate of Chinese cabbage with PAM and BP at 0.1% concentration, along with the CK.** The same letters above each bar indicate no difference between treatments determined by the Tukey’s HSD test at a significance level of 0.05 (*n* = 5).

Germination test was done to evaluate toxicity of PAM and BP treatment at each 0.1% rate for three days. After one day incubation, the germination rate was increased by an average of 13.6% in the Petri dishes treated with 0.1% PAM and BP compared to the CK (*P*< 0.05 in both cases; Figure 3).

After two days incubation, no difference in germination rate was found between BP and CK, but the germination rate with PAM treatment was 4.1% higher than others (*P* > 0.05). We conducted the same germination test with a lower concentration of PAM and BP at 0.05%; however, it did not show any difference with the CK (data not shown). There were also no differences among treatments after three days incubation (*P* > 0.05). We did not observe any negative effect of PAM and BP applications on the germination of Chinese cabbage. The similar results have been reported by Wallace and Wallace (1986) who evaluated the effects of PAM on the germination of tomato, cotton, and lettuce. Rawitz and Hazan (1978) also mentioned that PAM application has no toxicity or no negative effects on germination.

### Growth test for Chinese cabbage

Representative growth parameters for Chinese cabbage were produced as shown in Table 3. The values of leaf length from soils treated with PAM and BP were 8.0 and 11.3% higher than the CK, respectively, and those of leaf width were also 11.1 and 16.7% higher than the CK, respectively (*P* < 0.05 for both cases). However, no treatment effect on leaf number and chlorophyll content (in SPAD unit) was found. More importantly, the average value of dry weight from soils treated with PAM and BP was increased by 17.7% compared to the CK (*P* < 0.05). These findings confirmed the results from a large number of studies (Baasiri et al. 1986; Toyama et al. 1984; Woodhouse and Johnson 1991; Letey et al. 1992; Kim et al. 1998). Application of polymeric soil amendments commonly causes increasing plant growth due to improvement of soil physical properties, most likely related to water retention and plant-available water. It should be noted that many considerations including application rate, type of polymer, plant species, and soil types need to be addressed for crop productivity.

**Table 3:** **Leaf growth including number, length and width, SPAD readings, and dry weight of Chinese cabbage in loamy sand soils treated with PAM and BP at 200 kg ha** ^**-1**^ **, along with the CK**

Treatment				SPAD	Dry weight
	Number	Length	Width		g
		cm			
CK	33.3 ns^†^	19.6 b	12.0 b	42.4 ns	33.2 b
PAM	35.8 ns	21.3 a	13.5 a	44.3 ns	37.8 ab
BP	35.8 ns	22.1 a	14.4 a	45.6 ns	42.9 a

The same letters in table indicate no difference determined by the Tukey’s HSD test at a significance level of 0.05 (*n* = 3).

^†^ Not significant.

## Conclusions

This study was done to evaluate the effectiveness of commercially available PAM and synthesized BP originated from lignin, starch, acrylamide, and acrylic acid on soil erosion control, toxicity to plant and surrounding environments, and plant growth. Based on results from this study, the applications of PAM and BP do not have any negative effect. Partially, the synthesized BP treatment may be better for reducing nutrient loss from soils compared to commercial PAM. The aggregate stability was increased by 25.4 and 27.1% for soils treated with PAM and BP, respectively, compared to the CK. Moreover, the values of SS and turbidity were significantly reduced by up to 96.0 and 99.9%, respectively, compared to the CK. Moreover, the application of polymers didn’t show any negative effect or toxicity through seed germination or plant growth. The use of polymeric soil amendments may be an effective way to mitigate the current problems of soil erosion and nonpoint source pollution that we faced. In addition, the further research on BP synthesis using waste materials would be beneficial in the future.

## Authors’ original submitted files for images

Below are the links to the authors’ original submitted files for images. Authors’ original file for figure 1 Authors’ original file for figure 2 Authors’ original file for figure 3
