# Supplementary figures and images for: Commercial versus synthesized polymers for soil erosion control and growth of Chinese cabbage
Source: Springerplus. 2013 Oct 17;2(1):534. doi: 10.1186/2193-1801-2-534 (PMC4320192; doi:10.1186/2193-1801-2-534)

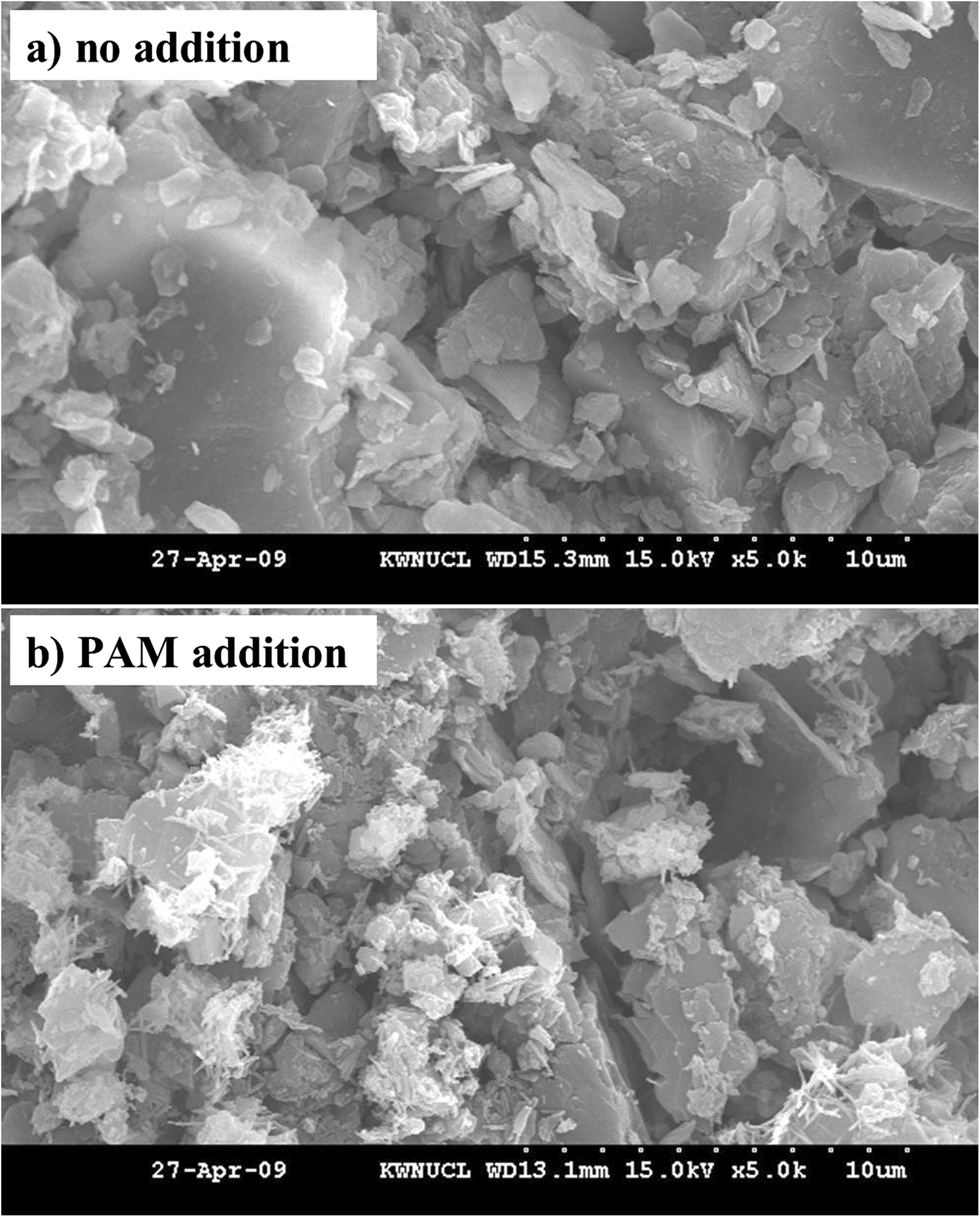

Supplement: Supplementary file 1 — Authors’ original file for figure 1 [file 40064_2013_1423_MOESM1_ESM.tif]

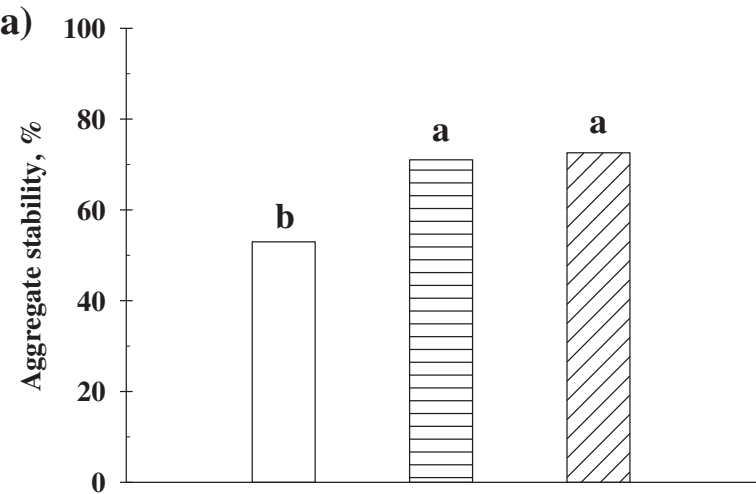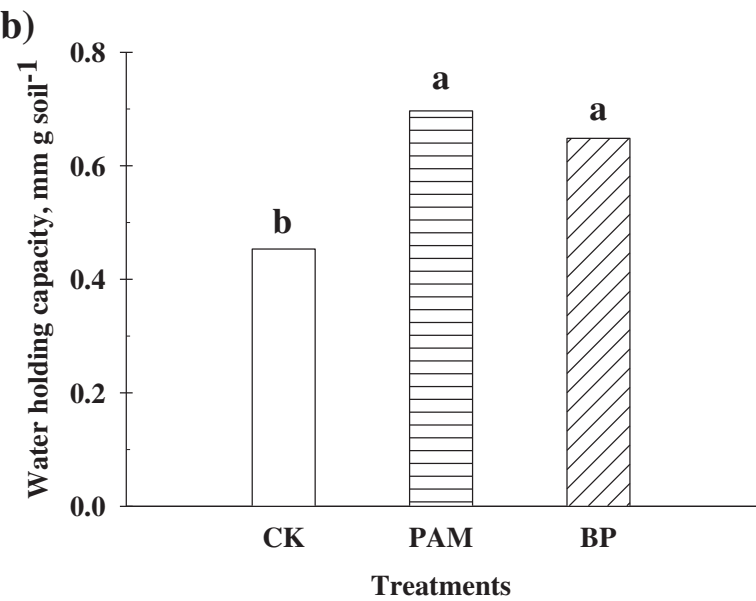

Supplement: Supplementary file 2 — Authors’ original file for figure 2 [file 40064_2013_1423_MOESM2_ESM.pdf]

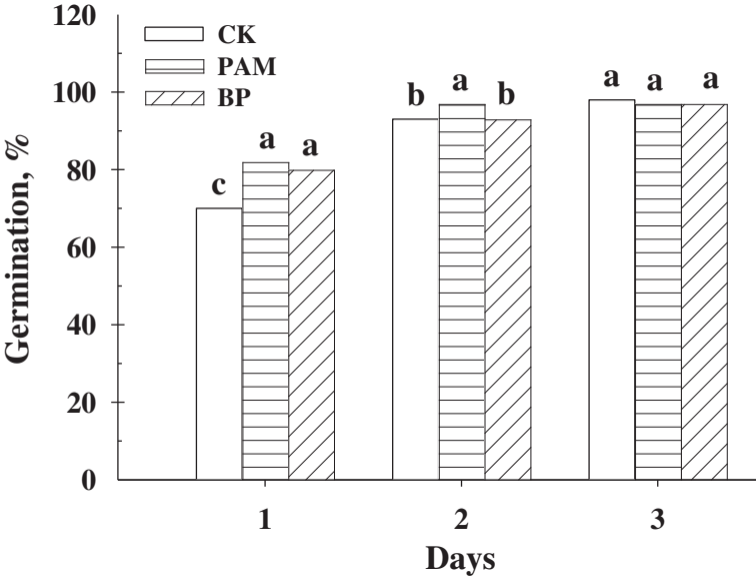

Supplement: Supplementary file 3 — Authors’ original file for figure 3 [file 40064_2013_1423_MOESM3_ESM.pdf]
